# Supplementary material for: Time-Dependent Reduction of Calcium Oscillations in Adipose-Derived Stem Cells Differentiating towards Adipogenic and Osteogenic Lineage
Source: Biomolecules. 2021 Sep 23;11(10):1400. doi: 10.3390/biom11101400 (PMC8533133; doi:10.3390/biom11101400)
Supplement: Supplementary file 1 [file biomolecules-11-01400-s001.zip › biomolecules-1318860-supplementary.pdf]

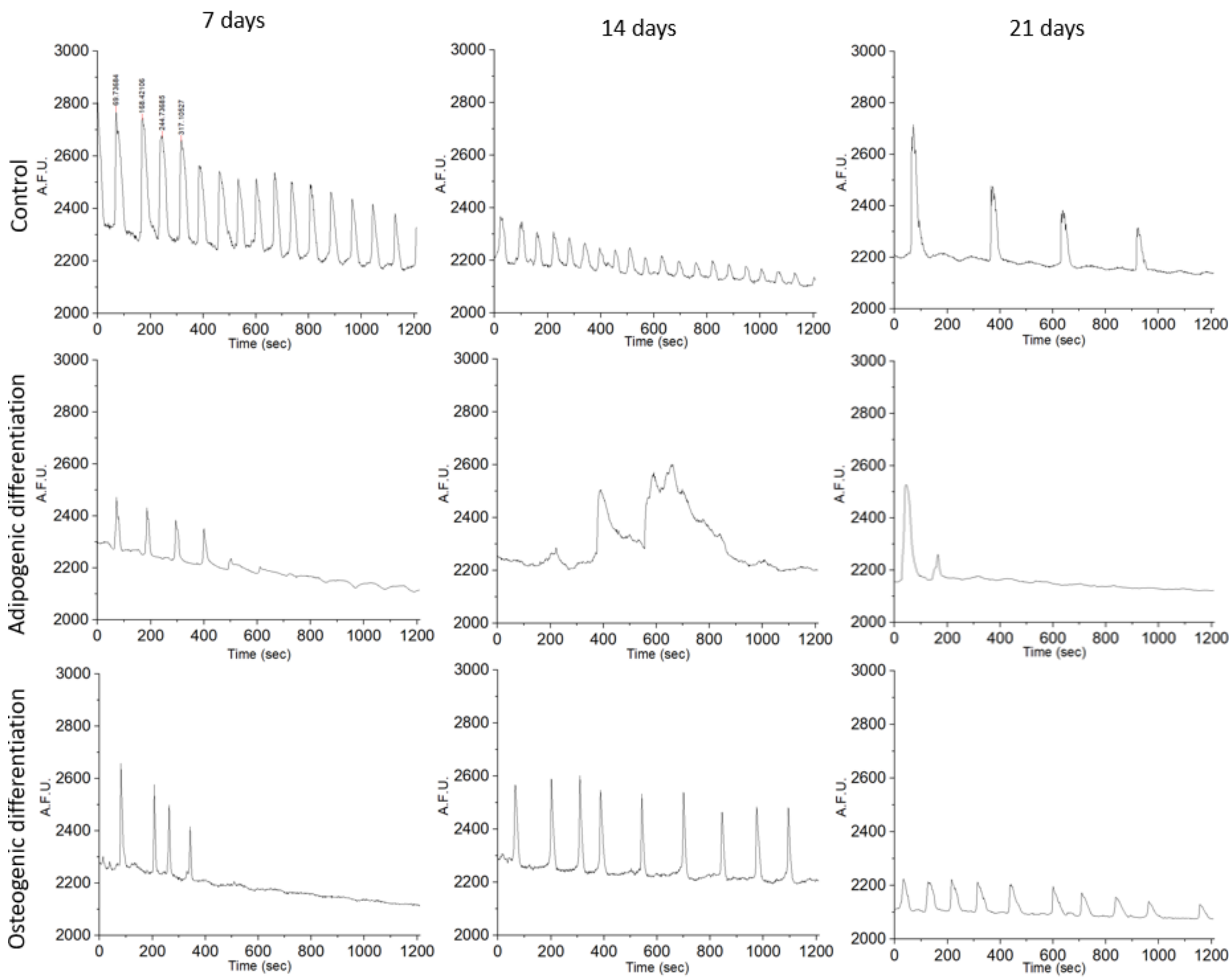

Figure SI 1. Representative traces of Ca^{2+} oscillations in each investigated condition. Each panel illustrates a representative example of single cell Ca^{2+} oscillations in each investigated condition. A ROI around the cell perimeter was defined and the fluorescence was measured for 20 minutes. These data sets were used to define the fraction of cells showing at least one oscillation and, in those oscillating, the interval between peaks as a direct measure of frequency. For the methodological details concerning the cell preparation, Fluo-4 staining, data acquisition and analysis see Section 2.1 to 2.5. Legend: A.F.U. = Arbitrary, Fluorescence Unites; sec = seconds.
